# On the measurement of healthy lifespan inequality

**DOI:** 10.1186/s12963-021-00279-8

**Published:** 2022-01-04

**Authors:** Iñaki Permanyer, Jeroen Spijker, Amand Blanes

**Affiliations:** 1 Centre d’Estudis Demogràfics, Carrer de Ca n’Altayó, Edifici E-2, Campus de la UAB, 08193 Cerdanyola del Vallès, Spain; 2 ICREA Research Professor. ICREA, Passeig Lluis Companys 23, 08010 Barcelona del Vallès, Spain

**Keywords:** Population health measures, Life expectancy, Health expectancy, Lifespan inequality, Socio-economic inequality, Spain, Education

## Abstract

**Background:**

Current measures to monitor population health include indicators of (i) average length-of-life (life expectancy), (ii) average length-of-life spent in good health (health expectancy), and (iii) variability in length-of-life (lifespan inequality). What is lacking is an indicator measuring the extent to which healthy lifespans are unequally distributed across individuals (the so-called ‘healthy lifespan inequality’ indicators).

**Methods:**

We combine information on age-specific survival with the prevalence of functional limitation or disability in Spain (2014–2017) by sex and level of education to estimate age-at-disability onset distributions. Age-, sex- and education-specific prevalence rates of adult individuals’ daily activities limitations were based on the GALI index derived from Spanish National Health Surveys held in 2014 and 2017. We measured inequality using the Gini index.

**Results:**

In contemporary Spain, education differences in health expectancy are substantial and greatly exceed differences in life expectancy. The female advantage in life expectancy disappears when considering health expectancy indicators, both overall and across education groups. The highly educated exhibit lower levels of lifespan inequality, and lifespan inequality is systematically higher among men. Our new healthy lifespan inequality indicators suggest that the variability in the ages at which physical daily activity limitations start are substantially larger than the variability in the ages at which individuals die. Healthy lifespan inequality tends to decrease with increasing educational attainment, both for women and for men. The variability in ages at which physical limitations start is slightly higher for women than for men.

**Conclusions:**

The suggested indicators uncover new layers of health inequality that are not traceable with currently existing approaches. Low-educated individuals tend to not only die earlier and spend a shorter portion of their lives in good health than their highly educated counterparts, but also face greater variation in the eventual time of death *and* in the age at which they cease enjoying good health—a multiple burden of inequality that should be taken into consideration when evaluating the performance of public health systems and in the elaboration of realistic working-life extension plans and the design of equitable pension reforms.

**Supplementary Information:**

The online version contains supplementary material available at 10.1186/s12963-021-00279-8.

## Background

When measuring population health across countries, life expectancy (LE) has become the most well-known and widely used indicator: its values are regularly reported by international institutions and National Statistical Offices worldwide. Despite its popularity, LE has two important shortcomings. First, its definition only takes into consideration the mortality of individuals, thus ignoring the health status of those who remain alive. Second, LE is simply an average that does not explain how the length of life is distributed across the members of a given population. To address these limitations, two important research avenues have evolved during the last decades. The first one has promoted the creation of ‘health expectancy indicators’ (HE) that measure the number of years that individuals are expected to live in ‘good health’ under prevailing mortality and morbidity conditions. These measures combine not only the quantity but also the quality (in terms of health) of the expected remaining years of life [1, 2]. The second one urges researchers and policy-makers alike to look beyond averages and assess how ‘unequal’ or ‘disperse’ length of life distributions are, i.e., to quantify the amount of ‘lifespan inequality’ (LI) existing in the ages-at-death distributions across population members [3, 4].

Surprisingly, key insights and contributions from these important research avenues have barely influenced each other over the years. While HE indicators have made the important distinction between ‘quantity’ and ‘quality’ of years of life and LI measures have separated ‘efficiency’ (i.e., average achievement) from ‘equality’, these two important analytical axes have not been considered simultaneously. The main proposal of this paper is to bring together these two strands of research into a coherent whole to obtain a deeper and more comprehensive understanding of contemporary population health dynamics. To attain this goal, we introduce the concept of ‘healthy lifespan inequality’ (HLI), which is designed to investigate the extent to which healthy (and unhealthy) lifespans are unequally distributed across population members. Rather than looking at the distribution of complete lifespans, we suggest partitioning the latter in the periods that individual spend across health states and investigating how these periods are distributed across individuals.

**Fig. 1:**
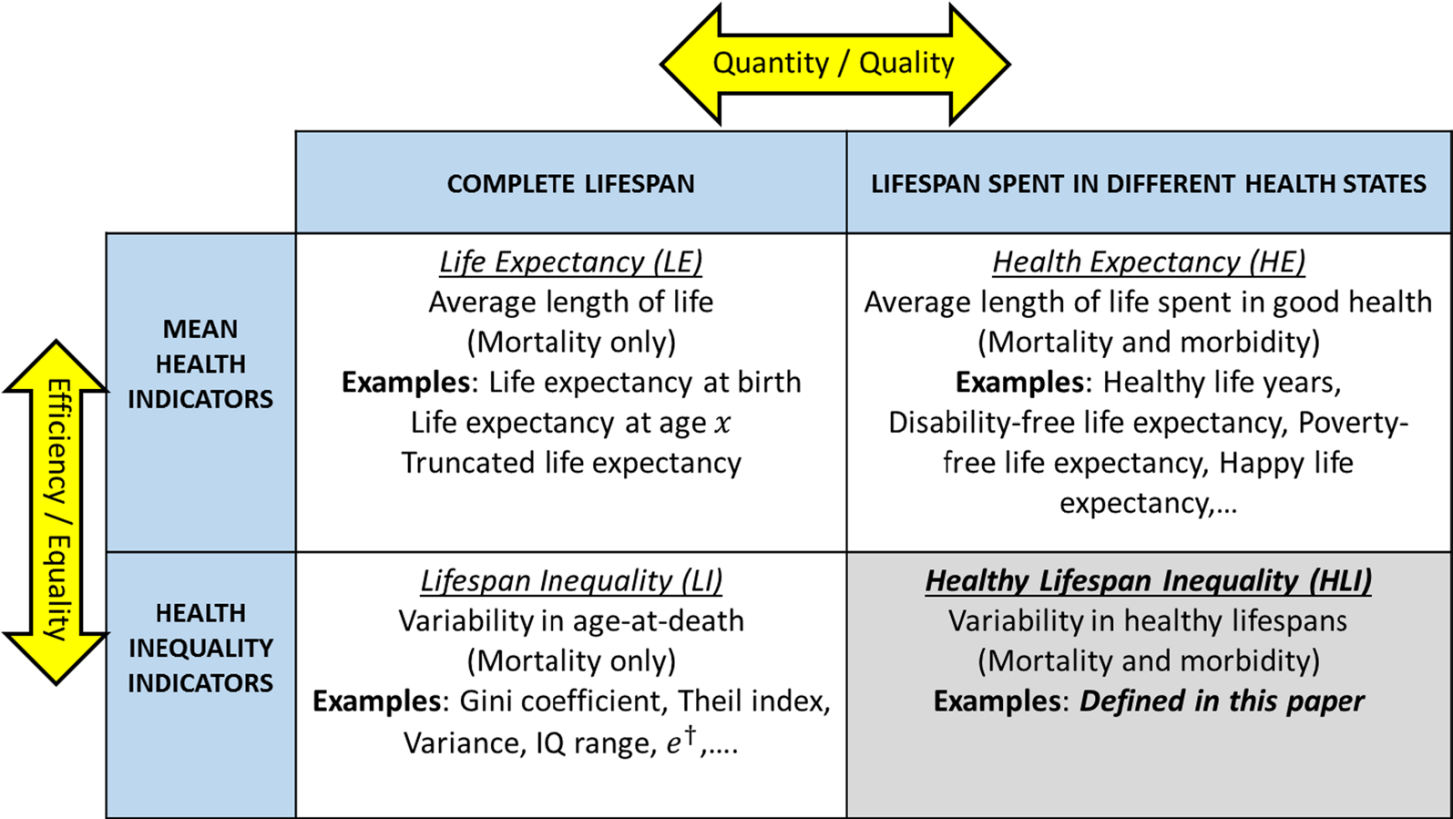
Summary diagram organizing population health indicators along the ‘quantity/quality’ and ‘efficiency/equality’ axes. *Source* Authors’ own elaboration

These different ideas are schematically represented in Fig. 1, in which the white cells show information and examples on existing families of population health indicators, and the shaded cell does so for the new family of indicators we propose to investigate here. The health outcome of the indicators included in the first column of Fig. 1 is “individuals’ longevity”, while the outcome of those in the second column is “individuals’ healthy longevity” (i.e., the number of years individuals live in good health). The indicators in the first row measure the mean of the distribution (i.e., they are an average) while those in the second row measure the variability/inequality in the distribution. The main contribution of this paper is to highlight that, currently, there are no indicators fitting in the second row and second column of the table, i.e., measuring the variability in individuals’ healthy lifespans. This is what the new HLI indicators are aiming at.

There are many reasons why healthy lifespan inequality can be considered a fundamental quantity in health research that should be reported alongside other well-known mortality and morbidity summary indicators. Population health means more than simply averting death, and societies are concerned not only about average levels of disease and/or disability, but also in the ways in which the latter are distributed. Larger levels of HLI indicate greater heterogeneity in underlying population health, an issue that can have implications both at the micro and at the macro level. At the individual level, HLI indicators measure uncertainty in the timing of disease, disability or physical limitation onset—the latter being key events with a strong and enduring impact on individuals’ well-being—with potentially important effects on individuals’ decision-making. At the macro level, HLI are appealing, simple measures of population health that might arguably be more meaningful than their LI counterparts: while the former look at the distribution of a normatively desirable quantity (‘years spent in good health’) the latter complicate matters by including quantities that might not be universally desirable (e.g., ‘years spent in very bad health’).

To illustrate the usefulness of the suggested approach, in this paper we investigate how LE, HE, LI and the new HLI indicators behave across sex and education groups in contemporary Spain.

## Methods

### Overview

**Fig. 2:**
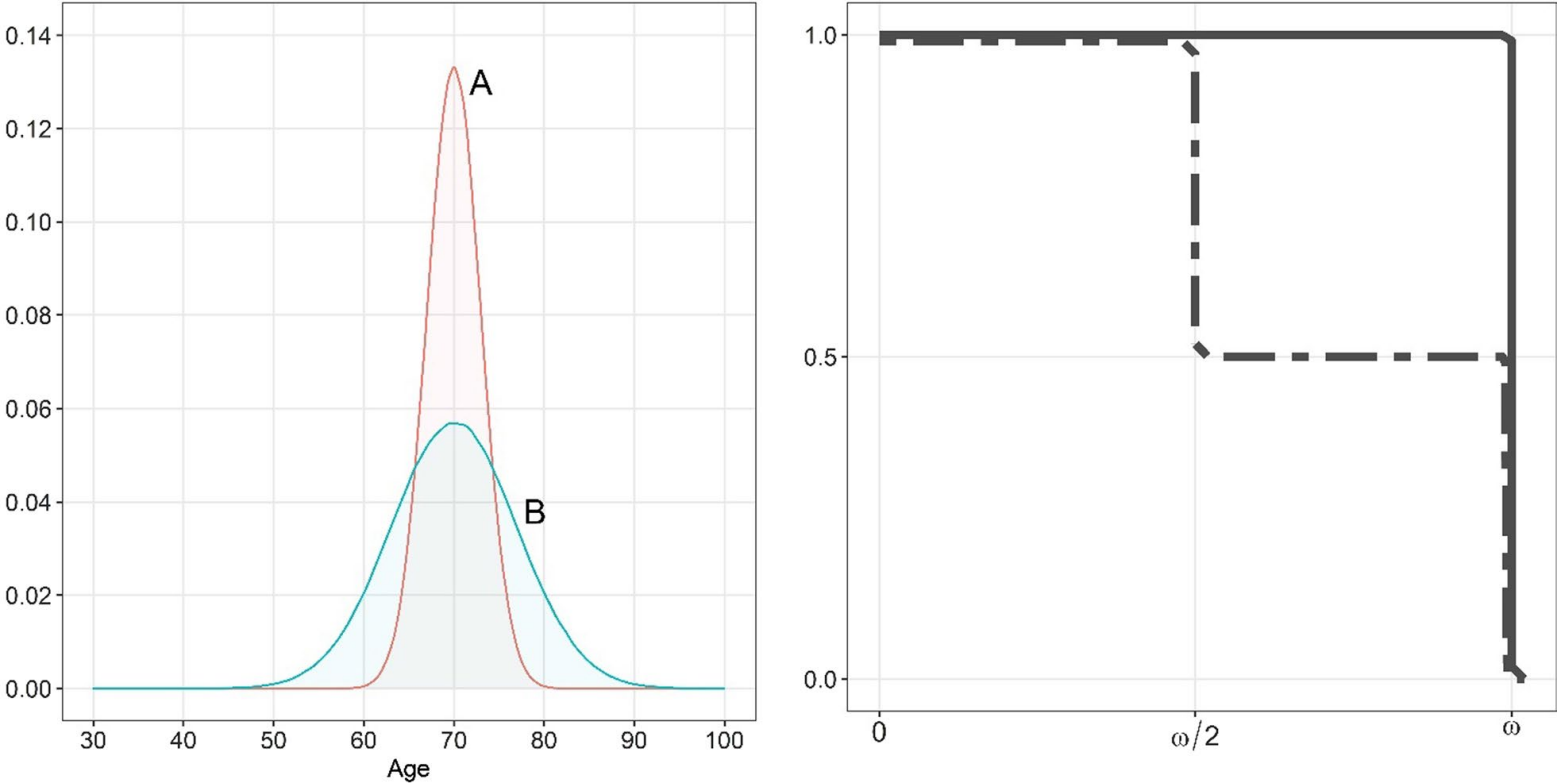
Hypothetical health distributions. Left panel: age at disability onset distributions for populations **A** and **B**; Right panel: survival and health-corrected survival curves (in continuous and dashed lines, respectively) for population **C**. *Source*: Authors’ own elaboration

Consider three hypothetical population health distributions. In the first one (*A*), individuals start developing a chronic disabling disease around the age of 70, and the variability in such ages is not very large. In the second one (*B*), the average age at which individuals start developing diseases is also 70, but the variability is much larger. While both *A* and *B* have 70 years of healthy life expectancy at birth—and are therefore indistinguishable for any HE indicator—the extent of health inequality in *B* is much larger than in *A.* Consider now a third hypothetical society *C* in which all individuals die when they approach the age *ω*, but in which one half of its members enjoy a perfect health until they die and the other half spends half of their lifetime in perfect health and the remaining half in very limiting health conditions (see right panel in Fig. 2). Despite the rampant health inequalities, current LI measures would conclude that length-of-life inequality in such society would be close to zero.

These examples suggest the need to complement currently existing measures of population health with indicators that simultaneously account for the quality (i.e. health status) of life years *and* the extent to which these years are equally distributed across individuals. This is what the new HLI indicators are meant for. To compute them we combine information on mortality and morbidity, and proceed in two steps. First, we estimate the distribution of age-at-disability onset for the population under study on the basis of the so-called ‘morbidity curve’ (see below), although other more sophisticated and data demanding methods can be implemented as well (see Discussion section). Second, we measure the amount of inequality in such distribution.

### Estimating health distributions

Similar to the Sullivan approach [5], we combine information on age-specific survival with information on the prevalence of functional limitation or disability to estimate the distribution of ages at which individuals cease to be in good health. As a measure of disability we use the Global Activity Limitation Index (GALI), originally designed and subsequently validated for health expectancy comparisons across Europe, and capturing long-term limitations (≥ 6 months) to perform daily activities due to health problems [6, 7]. The prevalence levels of disability by age, sex and education are smoothed using three-year moving averages to avoid some noisy fluctuations arising from the small sample size of some specific subgroups. We use a synthetic cohort approach in which its members are subject to current mortality and morbidity conditions along their lifetimes. Multiplying the ${l}_{x}$ column of the life table (showing the number of survivors at age $x$) by $1-{\pi }_{x}$ (the percent of population at age $x$ not limited to carry out daily activities) we obtain ${l}_{x}^{^{\prime}}$: the number of healthy survivors at age $x$. This is the so-called ‘morbidity curve’. From the ${l}_{x}$ and ${l}_{x}^{^{\prime}}$ columns we derive the standard ${d}_{x}$ and ${d}_{x}^{^{\prime}}$ distributions (i.e. number of deaths and individuals ceasing to be in good health between ages $x$ and $x+1$) defined as ${l}_{x}-{l}_{x+1}$ and ${l}_{x}^{^{\prime}}-{l}_{x+1}^{^{\prime}}$, respectively. The ages at which individuals die or cease to be in good health are thus described by the $\left\{{d}_{x}\right\}$ and $\left\{{d}_{x}^{^{\prime}}\right\}$ distributions.

### Inequality measurement

Once the age-at-death and age-at-disability-onset distributions have been estimated, we proceed to measure the corresponding levels of inequality. Many indicators are currently available [8], and all of them are highly correlated [4, 9]. To present our findings we use the Gini coefficient, a measure of inequality taking values between 0 (perfect equality) and 1 (maximal inequality). This is a frequently used indicator in studies of income or length-of-life inequality [3, 8, 9]. Other well-known inequality measures have been used as robustness checks.

### Other population health measures

The other population health measures used in this paper are standard LE, HE and LI indicators. LE measures the average time individuals are expected to live under the mortality conditions prevailing in a given year, while HE measures how much of that time is spent in good health. The latter is estimated using the Sullivan method [5], with the GALI indicator as a measure of disability prevalence. To measure LI, we apply the Gini index to the age-at-death distribution of the life table (i.e. the $\left\{{d}_{x}\right\}$ column). Observe that the only difference between LI and HLI indicators is that the former are based on the $\left\{{d}_{x}\right\}$ distribution, while the latter are based on the $\left\{{d}_{x}^{^{\prime}}\right\}$ distribution.

### Data

We use the mortality microdata files by educational attainment for those who died after age 35, which are publically available from the Spanish Statistical Office (INE). INE used a matching algorithm linking registered deaths to population databases, including censuses, municipal population registers, the ministry of education, and the Public State Employment Service [10], to obtain the deaths according educational attainment. The INE also provides the total population broken down by sex, age, and educational attainment, which are required for the denominators of our mortality indicators. With these registers, we can determine mortality levels across three education groups (Less than Primary, Primary, Secondary or more), and for women and men separately.

The GALI is obtained from the 2014 and 2017 Spanish National Health Surveys (SNHS). Specifically, respondents are asked if they have for the past six months or more been limited in ‘carrying out usual activities due to health problems’ [11]. We consider someone as disabled when they respond to be either ‘severely limited’ or ‘limited but not severely’. Data for both years are pooled and prevalence rates are generated by age, education and sex. Ages were bottom-truncated at 35 because of the low number of respondents in the lowest educational category (< 100) and to allow virtually all individuals to complete their formal education. Ages were also top truncated at 85 because of lacking representative morbidity data at higher ages for all education groups. This means that all LE, HE, LI and HLI indicators will be based on the 35–85 age range.

## Results

**Fig. 3:**
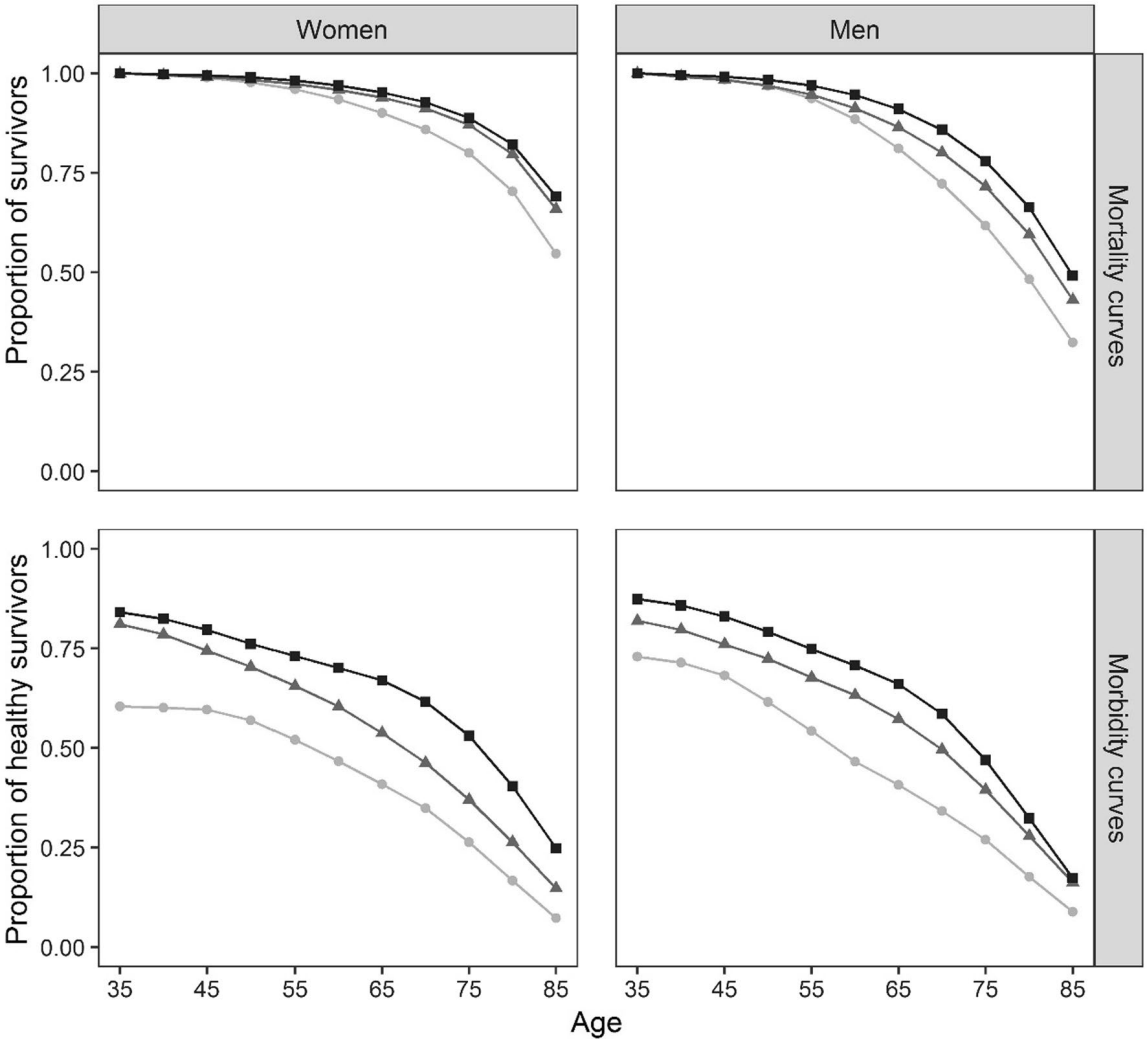
Synthetic cohort survival (top row) and healthy survival (bottom row) curves for women (first column) and men (second column) across education groups for ages 35–85, Spain 2014–17. *Source*: Authors’ calculations based on INE’s 2014–17 death files and the 2014 and 2017 SNHS

Figure 3 plots the estimated survival (top row) and healthy survival (bottom row) curves for ages 35–85 by sex and level of education for Spain in 2014–17. Among women, there are relatively small mortality differences across education groups (see upper left panel in Fig. 3). In contrast, the survival curves by educational attainment are further apart from each other among men (upper right panel), thus indicating a steeper mortality gradient across them. As regards the healthy survival curves, we observe the expected pattern: they decline with age and they are more favorable for the higher educated groups (see bottom row in Fig. 3). Both for women and for men, the declines of the healthy survival curves are more pronounced than their ‘survival-only’ counterparts. Such steeper decline suggests that the distributions of ages at physical limitation onset are more unequally distributed than the corresponding age-at-death distributions – an issue we will now quantify.

Table 1 presents the values of the mean health and health inequality indicators associated to the survival curves shown in Fig. 3. In the upper left panel we show truncated LE indicators, which should be interpreted in relative terms with respect to the maximal upper bound of 85–35 = 50 years (i.e., the 46.9 years of truncated life expectancy for highly educated women mean that this group attained 94% (100*46.9/50) of the maximal longevity potential). As expected, among both sexes higher educated groups are more longevous than less educated ones. Additionally, women are expected to live longer than men: the overall truncated LE are 46.4 and 43.1, respectively. Inspecting the levels of health expectancy (upper right panel), we observe that higher educated men and women are expected to live in good health for longer than their lower educated counterparts. Furthermore, the differences in HE across groups are much larger than the differences in LE. While longevity ranges between 44.5 and 46.9 for women and 40.4 and 44.3 for men, HE ranges from 22.4 to 34.0 for women and from 24.0 to 33.5 for men. The female advantage in LE disappears when considering HE indicators. Regarding lifespan inequality, it decreases with increasing education for both women and men. We also observe that LI is higher among men, overall and across all education groups (see bottom left panel). Finally, as regards the new healthy lifespan inequality indicators (see bottom right panel), we observe that:

(i) HLI are substantially larger than their LI counterparts, with the former being, on average, 50% larger than the latter.
(ii) HLI decreases with increasing education for both sexes.
(iii) Sex differences are not very large, but HLI indicator values tend to be somewhat higher for women.

**Table 1:**
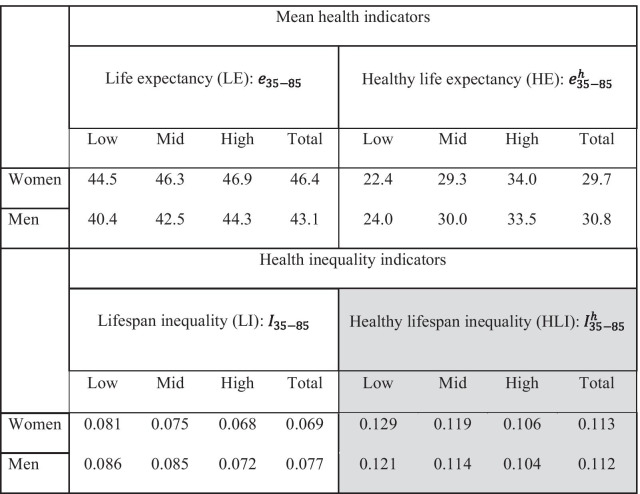
Life expectancy, healthy life expectancy, lifespan inequality and healthy lifespan inequality indicators for women and men aged 35–85 across education groups in Spain 2014–17. Morbidity measures are based on the GALI index and inequality is measured with the Gini coefficient. Shaded cells highlight the new HLI indicators proposed in this paper. *Source*: Authors’ calculations based on INE’s 2014–17 death files and the 2014 and 2017 SNHS

To ensure that our findings are not contingent upon arbitrary methodological choices, we have performed several robustness checks. First, we have re-calculated all our LI and HLI indicators for the different groups using alternative inequality measures, like the Gini mean difference, the Theil index or the Coefficient of Variation. All correlations among pairs of indicators are above 0.94. Second, we have used alternative measures to define less-than-good health, namely ‘self-assessed health’ and ‘ability to climb 12 stairs’. While different disability measures generate different values for the HE and HLI indicators, the substantive findings of this study remain unaltered. All robustness check calculations are shown in the Additional File 1.

## Discussion

This paper suggests incorporating the new family of ‘healthy lifespan inequality’ indicators into the list of measures that are regularly reported to monitor population health. To the extent that one is interested in measuring (1) how long individuals are expected to live, (2) how much of that time is spent in good health, and (3) how heterogeneous the distributions of lifespans can be, it is only natural to be also interested in the heterogeneity of the distribution of healthy lifespans. Applying the traditional LE, HE, LI together with the new HLI measures across education groups and sexes in contemporary Spain, several interesting patterns can be identified. In line with previous studies [12–14], we observe that education differences in HE are substantial and greatly exceed differences in LE, a well-known pattern also observed across countries (i.e. international differences in LE being smaller than international differences in HE [7]). The female advantage in LE disappears when considering HE indicators, overall and across education groups. The observed social patterning in LI coincides with recent studies suggesting that socio-economically advantaged groups tend to exhibit lower levels of lifespan inequality, and that the latter is systematically higher among men [4, 15–20].

The observed levels of LI dwarf when compared to their HLI counterparts: the new indicators suggest that the variability in the ages at which daily activity limitations start can be substantially larger than the variability in the ages at which individuals die (increases by a factor of 1.5). Our findings indicate that the levels of HLI are slightly higher for women – a result that coheres with the mortality advantage and morbidity disadvantage of women vis-à-vis men [21]. Importantly, HLI increases with decreasing educational attainment, both for women and for men. Recently, it has been suggested that because low-SES individuals tend to live shorter lives and face greater uncertainty in the age at which they will die than high-SES individuals, they are exposed to a ‘double burden of inequality’ [4, 18]. In light of our findings, where low-educated individuals are additionally expected to have shorter healthy lifespans and face greater uncertainty in the age at which they will start experiencing physical limitations, one could argue they are indeed exposed to a ‘quadruple burden of inequality’.

To our knowledge, this is the first study proposing measures to assess the extent to which the years spent in good health are (un)equally distributed across individuals. Previous analyses have investigated differences in health expectancies across subnational populations (e.g. comparing HE across women and men, socio-economic groups and/or racial groups [12–14]). While somewhat related, the two approaches are fundamentally different: the latter compares levels of HE across a closed list of pre-specified groups, and the former investigates variability in healthy lifespan distributions across individuals. These approaches echo a two-sided debate of the early 2000s, when the World Health Organization (2000) recommended going beyond group-based mean comparisons [22, 23] and incorporate individual-based data in the analysis of health inequalities [24, 25]. Since the turn of the century, the concept of inter-individual inequality has gained traction, thus favoring the spread of a bourgeoning literature on lifespan inequality. Yet, these contributions are based, implicitly or explicitly, on the assumption that ‘longer lives are normatively preferable to shorter ones’ – a gross simplification that greatly facilitates the construction of the corresponding LI measures but that might be hardly tenable in many circumstances. Confronted with the choice between ‘a prolonged yet unhealthy life’ and ‘a shorter but fully healthy life’, it is not clear that the former would be universally chosen in favor of the latter [26, 27]. The inclusion of any year of life irrespective of the health conditions in which that year is lived into standard lifespan inequality measures can muddy the waters in regard to the interpretation of their values. These conceptual problems are sidestepped by our HLI measures, which only take into consideration the variability in healthy lifespans.

In this paper we have focused our attention on the distribution of healthy lifespans, but it could be equally reasonable to look at the distribution of unhealthy lifespans. Both approaches are interesting in their own right, and it is not a priori clear what the relationship between the two approaches could be. One could easily imagine hypothetical scenarios with low levels of healthy lifespan inequality (i.e., everyone enjoying the same number of healthy years) but high levels of “unhealthy lifespan inequality” (i.e., large differences in the number of years individuals spend in bad health) or vice versa. These extremely interesting questions will be investigated in future research.

The new indices hold promise to be an important complement to traditional LE, HE and LI measures, which, on their own, do not explain the whole story and might lead to the elaboration of unfair or misinformed policies. Inter alia, HLI indicators can be crucial for the design of equitable pension schemes and retirement policies that are sensitive to the underlying heterogeneity in the population, and for the public provision of medical care (especially at advanced ages). From a public health policy perspective, larger HLI might be indicative of a worsening state of affairs across or within socially relevant groups – a cause of legitimate ethical concern, especially when social patterning in health is attributable to preventable causes. Finally, studying healthy lifespan variation can enrich the longstanding ‘compression/expansion of morbidity’ debate, which aims at understanding whether prevalence of morbid conditions accord with or diverge from trends in mortality [28, 29]. Since its inception more than 30 years ago, the contrasting hypotheses in this debate have been mostly tested by comparing LE with HE indicators (i.e., inspecting trends in *average* years of life vis-à-vis trends in *average* years in good health [1, 2, 7]). Yet, the original formulation of the compression of morbidity hypothesis [28]—which was stated in terms like ‘compression into a shorter span between the age of disability onset and death’, or ‘rectangularization of the morbidity curve’ – naturally lends itself into an inspection of HLI trends to test its validity. There are compelling reasons to believe that going beyond such ‘averages comparison’ by taking into consideration the entire distribution of ages at which death and diseases occur can throw considerable light into a long-lasting debate with crucial implications for understanding the development of human health and the performance of health systems.

This study has several limitations. First, our method to estimate healthy lifespan distributions is based on simplistic and somewhat unrealistic assumptions. Following our approach (in which the individuals of a fictitious cohort are subject to the current mortality and morbidity conditions throughout their lifetimes), we are implicitly assuming that there is no possibility of recovery from disease/disability, and that the risk of mortality is the same for healthy and unhealthy individuals. These are exactly the same limitations assailing the Sullivan method [5] that is commonly used to estimate HE indicators. Despite these shortcomings, the method has succeeded in becoming the workhorse of ‘health expectancy studies’ owing to its simplicity and applicability in a wide range of geographical, temporal and conceptual settings [1]. Some studies have shown that, under mild regularity conditions, Sullivan’s method is generally acceptable for monitoring long-term trends in HE [30]. In future research, it would be desirable to apply other methods to estimate healthy lifespan distributions more realistically. Some of these methods rely on longitudinal datasets that allow tracking individuals’ health trajectories over time and calculate transition probabilities across several health states (e.g. multistate life table or Markov chain techniques [1]). Unfortunately, the relative scarcity of longitudinal datasets across space and time limits the empirical applicability of such sophisticated methods.

Second, our approach to measure less-than-good health could be criticized on grounds of arbitrariness. ‘Health’ is a multidimensional and fuzzy concept whose measurement can be operationalized using other possible health outcomes. Additionally, there are several techniques to measure how such outcomes contribute to healthy/unhealthy life-years, using either dichotomous, ordinal or continuous scales [1, 2]. These well-known challenges have already been encountered by previous attempts to measure HE and compare its values across countries/over time [1] so they are not exclusive to the measurement of HLI indicators alone. To address them, it is important to use consistent definitions when making comparisons and, if feasible, use different health indicators to check the robustness of results. In our setting, the use of alternative conceptualizations of the concept of ‘disability’ besides the standard GALI indicator (i.e. self-perceived health or the ability to perform certain physical tasks) does not alter the substantive findings of the paper (see Additional File 1: Tables S3 and S4).

Third, our analyses are restricted to the ages ranging between 35 and 85 because of data limitation constraints. In all likelihood, the differences between women and men and across education groups would be altered if one considered all the ages above 35. Spain is among the world’s most longevous countries and many deaths and morbid conditions occur above age 85. Despite the limitations of our dataset, a clear education gradient emerges and the values of traditional LE, HE and LI indicators go in the expected direction. Thus, the quality of the dataset should be good enough to illustrate the usefulness of the new HLI indicators proposed in the paper.

## Conclusions

The previous limitations notwithstanding, the results of this study suggest that ‘healthy lifespan inequality’ is a substantively meaningful concept, which can be easily implemented in practice. The suggested approach is an invitation to go beyond country-level averages and explore not only the mean number of years individuals are expected to live in good health, but also the patterns in which these healthy years are distributed among them. Future research could determine whether the socio-economic health patterning we observe in contemporary Spain also extends to other countries around the world. In addition, exploring whether the factors that drive changes in HLI indicators are the same as those influencing the behavior of standard LE, HE and LI indicators can be a fruitful avenue of research to improve our understanding of contemporary health dynamics.

The HLI indicators uncover new layers of health inequality that are not observable with currently existing methods. In Spain, low-educated individuals tend to not only die earlier and spend a shorter portion of their lives in good health than their highly educated counterparts, but also face greater variation in the eventual time of death *and* in the age at which they cease enjoying good health—a multiple burden of inequality that should be taken into consideration when evaluating the performance of public health systems and in the elaboration of realistic working-life extension plans and the design of equitable pension reforms.

## Supplementary Information

**Additional file 1:** Supplementary Material with robustness checks.

## Data Availability

The data analyzed in this paper is available from the Spanish Statistical Office (INE; see https://www.ine.es).

## Abbreviations

LE: Life expectancy
HE: Health expectancy
LI: Lifespan inequality
HLI: Healthy lifespan inequality
INE: Spanish statistical office
SNHS: Spanish national health surveys
GALI: Global activity limitation index
