# Supplementary material for: On the measurement of healthy lifespan inequality
Source: Popul Health Metr. 2022 Jan 4;20:1. doi: 10.1186/s12963-021-00279-8 (PMC8725482; doi:10.1186/s12963-021-00279-8)
Supplement: Supplementary file 1 — Additional file 1: Supplementary Material with robustness checks. [file 12963_2021_279_MOESM1_ESM.docx]

**Supplementary Material**

*Robustness checks*

*1. Correlation among different inequality measures*

Many indicators exist to assess the extent of inequality in a given distribution. To test whether our findings are robust to the choice of alternative inequality measures we have performed the following exercise. We have computed lifespan inequality levels for the eight groups considered in this paper (three education groups + total, for women and men separately) using different inequality measures^1^ (the Gini coefficient (G), the Gini Mean Difference (G_a_, also known as ‘absolute Gini index’), the Theil index (T) and the Coefficient of Variation (CV)) and looked at pairwise correlations. The results, shown in table S1, suggest that the choice of alternative inequality measures does not modify our main conclusions. All correlation coefficients are very high (above 0.94).

| LI Corr | G | G_a_ | T | CV |
| --- | --- | --- | --- | --- |
| G | 1 |  |  |  |
| G_a_ | 0.99 | 1 |  |  |
| T | 0.94 | 0.97 | 1 |  |
| CV | 0.96 | 0.98 | 0.99 | 1 |

**Table S1.** Correlation matrix for different inequality measures for the length-of-life distributions. Source: Authors’ calculations based on INE’s 2014-17 death files and the 2014 and 2017 SNHS.

Repeating the same exercise for healthy lifespan inequality measures, we obtain the results shown in Table S2. Again, the choice of alternative inequality measures does not modify our main conclusions. All correlation coefficients are very high (above 0.94).

| HLI Corr | G | G_a_ | T | CV |
| --- | --- | --- | --- | --- |
| G | 1 |  |  |  |
| G_a_ | 0.96 | 1 |  |  |
| T | 0.94 | 0.99 | 1 |  |
| CV | 0.97 | 0.99 | 0.99 | 1 |

**Table S2.** Correlation matrix for different inequality measures for the healthy length-of-life distributions. Source: Authors’ calculations based on INE’s 2014-17 death files and the 2014 and 2017 SNHS.

*2. Using other health outcome measures*

The ages at which disability starts deteriorating individuals’ health crucially depend on the indicator that is chosen to measure non-fatal outcomes. To check if our findings are robust to the choice of alternative measures, we replicate our main results table (the one reporting the values of the LE, HE, LI and HLI indicators across education groups and sexes in contemporary Spain) substituting the GALI indicator used in the main text by two other health outcome measures. The first one is ‘self-perceived health’, a frequently used indicator for health that deals with the subjective assessment that a person makes about one’s own health state. It serves as an independent predictor for morbidity, health service use and mortality, and has often been used as an indicator to compute healthy life expectancy^2^. The individual’s status of self-perceived health is measured with the SNHS question ‘How is your health in general?’. For the purpose of the exercise the possible answers “very good”, “good”, “regular”, “bad” and “very bad” were dichotomised into “good health” (combining “good” and “very good” health) and “less than good health” (combining “fair”, “bad” and “very bad” health). The results for the ‘self-perceived less than good health’ indicator are shown below.

|  | Mean health indicators | | | | | | | |
| --- | --- | --- | --- | --- | --- | --- | --- | --- |
|  | Life expectancy (LE) | | | | Healthy life expectancy (HE) | | | |
|  |  |  |  |  |  |  |  |  |
|  | Low | Med | High | Total | Low | Med | High | Total |
| Women | 44.5 | 46.3 | 46.9 | 46.4 | 19.2 | 25.8 | 33.3 | 27.2 |
| Men | 40.4 | 42.5 | 44.3 | 43.1 | 21.2 | 27.2 | 32.6 | 28.8 |
|  | Health inequality indicators | | | | | | | |
|  | Lifespan inequality (LI) Gini | | | | Healthy lifespan inequality (HLI) Gini | | | |
|  |  |  |  |  |  |  |  |  |
|  | Low | Med | High | Total | Low | Med | High | Total |
| Women | 0.081 | 0.075 | 0.068 | 0.069 | 0.131 | 0.131 | 0.125 | 0.126 |
| Men | 0.086 | 0.085 | 0.072 | 0.077 | 0.137 | 0.128 | 0.109 | 0.121 |

**Table S3.** Life expectancy, healthy life expectancy, lifespan inequality and healthy lifespan inequality indicators for women and men aged 35-85 across education groups in Spain 2015. The disability measures are based on individuals’ self-perceived health and inequality is measured with the Gini coefficient. Shaded cells highlight the new HLI indicators proposed in this paper. Source: Authors’ calculations based on INE’s 2014-17 death files and the 2014 and 2017 SNHS.

As can be seen, the findings reported in the main paper and the ones reported in Table S3 essentially go in the same direction. (i) Differences in health expectancy across education groups are much larger than differences in life expectancy (always benefiting the highly educated), (ii) health expectancy indicators tend to be slightly better for men than for women, (iii) healthy lifespan inequality is much higher than lifespan inequality, (iv) healthy lifespan inequality decreases with educational attainment, and (v) the variability in age-at-disability onset tends to be slightly higher among women than among men.

The second measure for which we replicate the four health indicators is the capacity to go up and down a flight of 12 stairs uninterruptedly. Like the GALI, this measure is an indicator of disability or functional limitation^3^. Specifically, the respondent is asked if he or she has ‘difficulty to go up or down 12 stairs’. To calculate the health indicators the response category “no difficulty” was kept and “some difficulty”, “severe difficulty” and “I cannot do it by myself “ were collapsed into “with difficulty/can’t do it alone”. The results for the ‘climbing 12 stairs’ indicator are shown below.

|  | Mean health indicators | | | | | | | |
| --- | --- | --- | --- | --- | --- | --- | --- | --- |
|  | Life expectancy (LE) | | | | Healthy life expectancy (HE) | | | |
|  |  |  |  |  |  |  |  |  |
|  | Low | Med | High | Total | Low | Med | High | Total |
| Women | 44.5 | 46.3 | 46.9 | 46.4 | 27.6 | 35.1 | 38.8 | 35.0 |
| Men | 40.4 | 42.5 | 44.3 | 43.1 | 29.8 | 35.6 | 38.5 | 36.1 |
|  | Health inequality indicators | | | | | | | |
|  | Lifespan inequality (LI) Gini | | | | Healthy lifespan inequality (HLI) Gini | | | |
|  |  |  |  |  |  |  |  |  |
|  | Low | Med | High | Total | Low | Med | High | Total |
| Women | 0.081 | 0.075 | 0.068 | 0.069 | 0.128 | 0.107 | 0.090 | 0.102 |
| Men | 0.086 | 0.085 | 0.072 | 0.077 | 0.113 | 0.105 | 0.086 | 0.098 |

**Table S4.** Life expectancy, healthy life expectancy, lifespan inequality and healthy lifespan inequality indicators for women and men aged 35-85 across education groups in Spain 2015. The disability measures are based on individuals’ ability to climb 12 stairs without difficulty, and inequality is measured with the Gini coefficient. Shaded cells highlight the new HLI indicators proposed in this paper. Source: Authors’ calculations based on INE’s 2014-17 death files and the 2014 and 2017 SNHS.

Once again, the main findings of this paper are reproduced when using this alternative definition of physical limitation.

**References Supplementary Material**

1. Villar, A. Lectures on Inequality, Poverty and Welfare. *Lecture Notes in Economics and Mathematical Systems* 685, Springer (2017).
2. Croezen S, Burdorf A, van Lenthe FJ. Self-perceived health in older Europeans: Does the choice of survey matter? *Eur J Public Health* 2016; **26**(4): 686-92.
3. Latorre-Román PA, Laredo-Aguilera JA, García-Pinillos F, Soto-Hermoso VM, Carmona-Torres JM. Physical activity, weight and functional limitations in elderly Spanish people: the National Health Survey (2009–2014). *The European Journal of Public Health* 2018; **28**(4): 778-83.
